# Evaluation of [^11^C]-Methionine Positron Emission Tomography and Cerebral Blood Volume Imaging in the Diagnosis of Non-Contrast-Enhanced Gliomas

**DOI:** 10.3390/jcm14196777

**Published:** 2025-09-25

**Authors:** Naoya Imai, Hirohito Yano, Yuka Ikegame, Shoji Yasuda, Ryo Morishima, Soko Ikuta, Noriyuki Nakayama, Takashi Maruyama, Naoyuki Ohe, Morio Kumagai, Yoshihiro Muragaki, Jun Shinoda, Tsuyoshi Izumo

**Affiliations:** 1 Department of Neurosurgery, Chubu Medical Center for Prolonged Traumatic Brain Dysfunction, Chubu Neurorehabilitation Hospital, Minokamo 505-8503, Japan; hirohito@herb.ocn.ne.jp (H.Y.); ikegame-nsu@umin.ac.jp (Y.I.); r-morishima@air.ocn.ne.jp (R.M.); mor123155@yahoo.co.jp (M.K.); junshino@joy.ocn.ne.jp (J.S.); 2 Department of Neurosurgery, Graduate School of Medicine, Gifu University, Gifu 501-1194, Japan; yasuda.shoji.u3@f.gifu-u.ac.jp (S.Y.); nakayama.noriyuki.u4@f.gifu-u.ac.jp (N.N.); ohe.naoyuki.r3@f.gifu-u.ac.jp (N.O.); go-izumo@hotmail.co.jp (T.I.); 3 Department of Clinical Brain Sciences, Graduate School of Medicine, Gifu University, Gifu 501-1194, Japan; 4 Department of Neurosurgery, Tokyo Women’s Medical University, Tokyo 162-8666, Japantmaruyama@twmu.ac.jp (T.M.); ymuragaki@twmu.ac.jp (Y.M.)

**Keywords:** glioma, positron emission tomography, perfusion magnetic resonance imaging

## Abstract

**Background/Objectives**: Methionine (MET) positron emission tomography (PET) and cerebral blood volume (CBV) imaging provide complementary glioma assessment. This study compared MET and CBV across glioma subtypes defined by the 2021 World Health Organization Classification. **Methods**: This retrospective study enrolled 106 patients (mean age 41.9 ± 12.4 years; 57 males) with MRI non-contrast-enhanced gliomas: 21 glioblastoma, isocitrate dehydrogenase (IDH)-wildtype (G); 50 astrocytoma, IDH-mutant (A); and 35 oligodendrogliomas, IDH-mutant, and 1p/19q-codeleted (O). Relative CBVs (rCBVs) were measured in VOI-T2 and VOI-MET, and the MET tumor-to-normal (T/N) ratio was calculated. **Results**: MET and rCBV were significantly correlated (r = 0.5, *p* < 0.001); rCBV was higher in MET-positive tumors and predicted MET accumulation (area under the curve [AUC] = 0.72, cutoff = 2.99). In VOI-T2, rCBV and MET T/N ratio were the highest in G and lowest in A (*p* < 0.001). Receiver operating characteristic analyses showed no overall significant difference between MET and rCBV for differentiating G/A/O, but rCBV trended toward higher AUC values in key distinctions, such as G (0.736 vs. 0.612) or grade 4 (0.718 vs. 0.617). The increase in rCBV within the MET-positive region (VOI-MET/VOI-T2 rCBV ratio) was significantly higher in A (119.8%, *p* = 0.002) than in the other groups (*p* = 0.01). **Conclusions**: rCBV differentiated glioma subtype with accuracy comparable to MET and could predict MET accumulation. However, its reliability for identifying MET-positive regions varied by subtype, being useful in A but limited in O. Recognizing these subtype-specific differences, rCBV can serve as a practical tool for evaluating non-contrast-enhanced gliomas.

## 1. Introduction

Gliomas are highly malignant primary tumors that arise in the central nervous system. In 2021, the World Health Organization (WHO) classification transitioned from a pathology-based diagnosis to one that emphasizes genetic alterations, categorizing adult-type diffuse gliomas into three distinct types. Various imaging modalities have been employed for gliomas. Glioma imaging has evolved to encompass a variety of MRI and PET applications, and more recently, multimodal approaches that integrate these modalities [1,2]. [^11^C]-methionine (MET) positron emission tomography (PET) has been utilized to evaluate the extent of tumor invasion, proliferation, and prognosis and differentiate between radiation necrosis and recurrence [1,2,3,4,5,6,7,8]. Among these, MET accumulation has three possible explanations: active transport, passive diffusion, and stagnation. MET is actively transported into tumor cells via an energy-independent L-type amino acid transporter system, similar to the sodium-dependent transporter system. While active transport reflects cell proliferation and tumor malignancy, passive diffusion is associated with disruption of the blood–brain barrier (BBB). Stagnation depends on blood volume. However, MET PET is not versatile because facilities with cyclotrons are limited.

In contrast, cerebral blood volume (CBV) obtained from dynamic susceptibility contrast (DSC) perfusion-weighted imaging (PWI) has been reported to aid in assessing tumor invasion, identifying genetic mutations, and distinguishing radiation necrosis from other brain tumors [9,10,11,12,13,14,15,16]. CBV predicts malignancy by reflecting mitotic activity, vascular permeability, and increased angiogenesis [9,11]. Contrast-enhanced magnetic resonance imaging (MRI) is practically mandatory for diagnosing brain tumors; therefore, DSC-PWI is a straightforward addition that does not require additional invasiveness.

The effectiveness of CBV imaging and MET PET has been reported, each employing a different mechanism for diagnosing gliomas. Previous studies combining MET and CBV have focused on the correlation between the two and discussed the differentiation of low-grade gliomas, high-grade gliomas, radiation necrosis, and the extent of tumor invasion of high-grade gliomas [17,18,19,20,21]. However, these studies included relatively small cohorts and did not separate cases based on the presence or absence of contrast-enhanced lesions, tumor grade, or molecular diagnosis. Since the adoption of the 2021 WHO classification, differentiating non-contrast-enhanced gliomas, including astrocytoma, oligodendroglioma, and glioblastoma, with MRI has not always been straightforward, as all isocitrate dehydrogenase (IDH) wild-type tumors are diagnosed as grade 4, even without contrast enhancement.

The novelty of this study lies in it being one of the largest series to date focusing specifically on non-contrast-enhanced gliomas classified under the 2021 WHO criteria. By diagnosing glioblastoma, IDH-wildtype (G), astrocytoma, IDH-mutant (A), and oligodendroglioma, IDH-mutant and 1p/19q-codeleted (O) using MET PET and CBV imaging, and by clarifying whether the reported correlation between MET and CBV applies uniformly across all glioma subtypes or varies by histological type, this study aimed to provide clinically relevant insights and validate the value of CBV imaging as a more widely accessible alternative to MET PET.

## 2. Materials and Methods

### 2.1. Patients

A retrospective study was conducted on patients who underwent MET PET and MRI (T2WI, contrast-enhanced T1WI, and DSC-PWI) on the same day at our institution between May 2012 and December 2022. All non-contrast-enhanced adult-type diffuse supratentorial gliomas were diagnosed according to the 2021 WHO Classification, as emphasized in recent studies [22]. All patients underwent biopsy or surgical resection, and histopathological confirmation was obtained before inclusion. IDH status was examined via immunohistochemistry using anti-IDH1 R132H monoclonal antibodies and anti-IDH2 R172 antibodies on formalin-fixed paraffin-embedded sections, following previously reported methods [6]. Immunoreactivity was considered positive when strong and diffuse staining of the tumor cells was observed. Fluorescence in situ hybridization was used to assess the loss of heterozygosity in 1p and 19q. The exclusion criteria included any MRI-enhanced lesion (including faint or patchy enhancement), infratentorial location, unknown genetic status, age < 18 years, or poor imaging quality. Poor imaging refers to difficulty in calculating CBV or fusing images due to motion artifacts or a lack of PWI data.

This study was approved by our hospital’s Institutional Ethics Review Board (Approval No. 2024-16) and conducted in accordance with the principles of the 1964 Declaration of Helsinki, its subsequent amendments, and equivalent ethical standards. The need for informed consent was waived owing to the retrospective design of the study. Patients could opt out after reviewing the disclosure document on the website (https://cnrh.jp/wp/wp-content/uploads/2024/08/optout20240819.pdf, accessed on 22 September 2025).

### 2.2. MRI Procedure

All patients underwent MRI using the Achieva 3.0-Tesla TX QD MRI system (Philips, Amsterdam, The Netherlands). T1WI and T2WI were acquired with the following parameters: repetition time = 2100/2500 ms, echo time = 9.5/230 ms, flip angle = 90°/90°, matrix size = 512 × 512/240 × 240, field of view = 230 × 230 mm/240 × 240 mm, and slice thickness = 5 mm. Patients were injected with 9 mL of a gadolinium-based contrast agent for contrast-enhanced sequences, followed by a 20 mL saline flush. DSC-PWIs were obtained simultaneously with the contrast injection using the following parameters: fast-field echo, echo-planar imaging, repetition time = 1000 ms, echo time = 35 ms, flip angle = 70°, matrix size = 128 × 128, field of view = 230 mm × 230 mm, 60 dynamics, and slice thickness = 5 mm.

### 2.3. PET Procedures

PET was performed using Eminence STARGATE (Shimadzu Corporation, Kyoto, Japan) equipped with a three-dimensional acquisition system. This system provided 99 transaxial images at 2.65 mm intervals, with an in-plane spatial resolution (full width at half maximum) of 4.8 mm. The axial images were aligned parallel to the canthomeatal line. MET tracers were injected at 3.5 MBq/kg, and tracer accumulation was assessed using the standardized uptake value.

### 2.4. Analysis Technique

**Figure 1:**
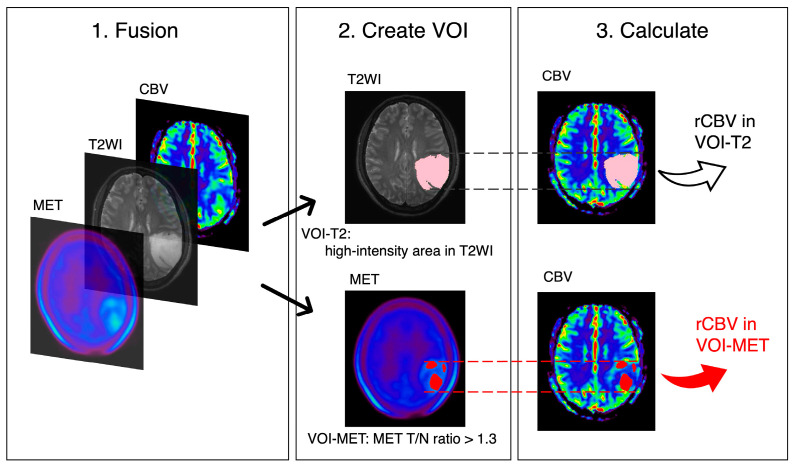
Volume of interest (VOI) placements. T2-weighted image (T2WI), methionine (MET) PET, and cerebral blood volume (CBV) maps are fused and reconstructed. rCBV values are calculated for VOI-T2 (high-intensity area in T2WI, pink) and VOI-MET (area with MET tumor to normal (T/N) ratio > 1.3, red).

All analyses were performed using the Dr. View/Linux software (R2.5.0AJS Corporation, Tokyo, Japan). The CBV maps were calculated from DSC-PWI. Voxel-wise changes in relative contrast agent concentration were determined by converting signal intensity–time curves into relaxation rate–time curves. CBV was estimated as the area under the relaxation rate–time curve [23]. T2WI, MET PET, and CBV maps were reconstructed and fused. High-intensity areas from the T2WI were semi-automatically drawn as the volume of interest (VOI)-T2. Ratios of tumor maximum standardized uptake value to normal frontal cortex standardized uptake value (T/N) were calculated from MET PET. Based on a previous report, VOI-MET was defined as the area of tumor parenchyma with the T/N ratio exceeded 1.3 [1,3,4]. Cases in which VOI-METs could be generated were considered to have MET accumulation. A neurosurgeon generated the T/N ratio, VOI-T2, and VOI-MET, without access to the patient’s data or study details. The relative CBVs (rCBVs: mean CBV in each VOI divided by the CBV of contralateral normal white matter) were calculated (Figure 1). These previously reported methods are considered accurate and universally applicable [19,20,21,23,24].

### 2.5. Data Analysis

The patient background, T/N ratio, and rCBV on VOI-T2/MET were compared based on MET accumulation, glioma subtype, and IDH status. Pearson’s chi-square test was used to analyze categorical variables. Mann–Whitney U and Kruskal–Wallis tests were applied to continuous variables. The Steel–Dwass test was used for multiple comparisons. Pearson’s correlation coefficients between the rCBV and the MET T/N ratio were calculated and plotted as regression lines. A paired two-sample *t*-test was used to compare the rCBV between VOI-T2 and VOI-MET. Receiver operating characteristic (ROC) curves were used to calculate cutoff values, sensitivity, specificity, and the area under the curve (AUC). The Youden index was applied to identify optimal thresholds, predict MET accumulation, and diagnose glioma subtypes using the rCBV and MET T/N ratio. DeLong’s test was performed to compare the two ROC curves. The EZR software (v. R 4.5.0, Saitama Medical Center, Jichi Medical University, Saitama, Japan, https://www.jichi.ac.jp/usr/hema/EZR/statmed.html, accessed on 22 September 2025) was used for DeLong’s test. The JMP statistical software (v. 17.1.0, SAS Institute Inc., Cary, NC, USA) was used for all other statistical analyses. Statistical significance was set at *p* < 0.05.

## 3. Results

**Figure 2:**
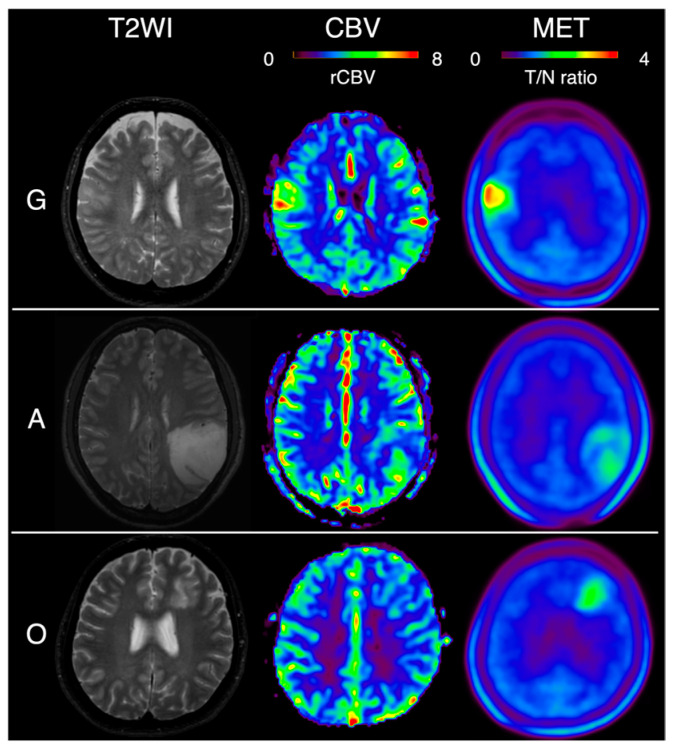
T2-weighted image (T2WI), relative cerebral blood volume (rCBV), and methionine (MET) PET imaging in typical cases of each glioma subtype. G: glioblastoma isocitrate dehydrogenase (IDH)-wildtype, A: astrocytoma, IDH-mutant, O: oligodendrogliomas, IDH-mutant, and 1p/19q-codeleted. Color map scales: CBV, rCBV-to-white matter ratio up to 8; MET, tumor-to-normal (T/N) ratio up to 4.

During the study period, 527 patients with glioma underwent preoperative PET and MRI. Pathological diagnoses were confirmed in 245 cases, and after excluding 139 patients based on the exclusion criteria, 106 patients were included in the analysis. Of these, 21 cases were G; 50 were A (with 8 cases of grade 2, 41 cases of grade 3, and 1 case of grade 4) and 35 were O (with 26 cases of grade 2 and 9 cases of grade 3). No pseudopalisading necrosis was observed. A summary of the 106 non-contrast-enhanced glioma cases is shown in Table 1. Representative images of each subtype are shown in Figure 2.

**Figure 3:**
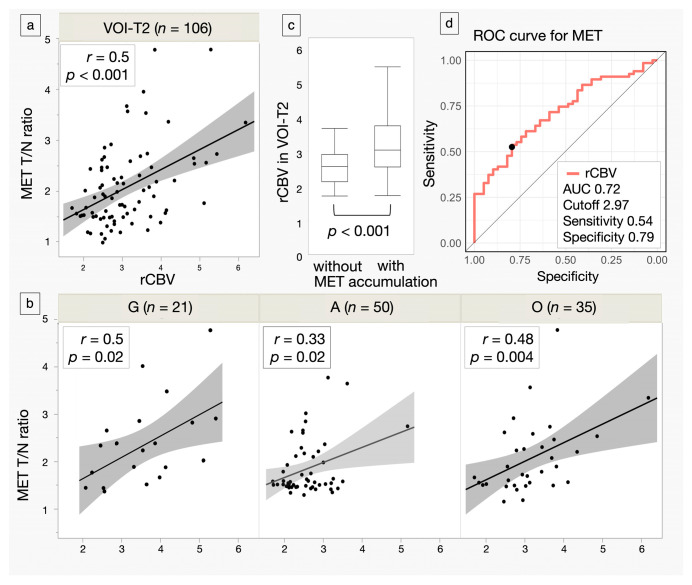
Scatter plots of methionine (MET) tumor-to-normal (T/N) ratio and relative cerebral blood volume (rCBV) in the volume of interest (VOI)-T2 and VOI-MET for all cases and each glioma subtype. Regression lines are shown with intensity reflecting correlation strength. N indicates the number of subjects. (**a**,**b**) Box plots comparing rCBV in the VOI-T2 for gliomas with and without MET accumulation. Lines within the boxes indicate the mean, while the boxes represent the interquartile range. (**c**) Receiver operating characteristic (ROC) curves show the area under the curve (AUC), cutoff value, sensitivity, and specificity of rCBV in detecting gliomas with MET accumulation. The closed circle denotes the Youden Index, representing the cutoff value. (**d**) G: glioblastoma, isocitrate dehydrogenase (IDH)-wildtype, A: astrocytoma, IDH-mutant, O: oligodendroglioma, IDH-mutant, and 1p/19q-codeleted.

The correlations between the MET T/N ratio and rCBV in VOI-T2 and VOI-MET are shown in Figure 3a,b. rCBV in VOI-T2 was significantly higher in patients with MET accumulation (3.22 ± 0.95) than in those without accumulation (2.56 ± 0.52, *p* < 0.001) (Figure 3c). The cutoff value for the presence or without of MET accumulation, obtained from the ROC curve using rCBV in VOI-T2, was 2.97, with a sensitivity of 53.7%, a specificity of 79.4%, and an AUC of 0.72 (Figure 3d).

**Figure 4:**
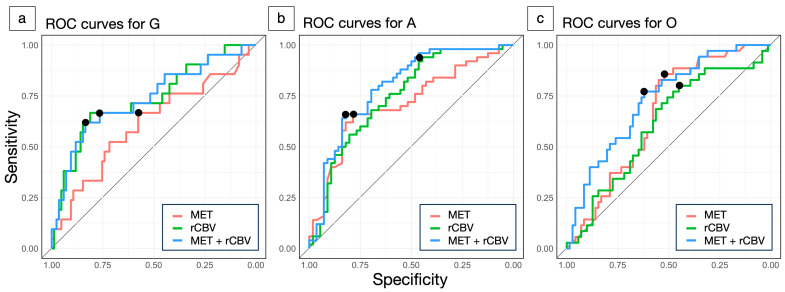
Receiver operating characteristic (ROC) curves for differentiating glioma subtypes using methionine (MET) tumor-to-normal (T/N) ratio (red), relative cerebral blood volume (rCBV) (green), and their combination (blue). Glioblastoma, isocitrate dehydrogenase (IDH)-wildtype (G) vs. others (**a**), astrocytoma, IDH-mutant (A) vs. others (**b**), oligodendrogliomas, IDH-mutant, and 1p/19q-codeleted (O) vs. others (**c**). The closed circle denotes the Youden Index, representing the cutoff value.

In the subtype comparison, the rCBV in VOI-T2 (G: 3.60 ± 1.0, A: 2.60 ± 0.60, O: 3.14 ± 0.89) and the MET T/N ratio (G: 2.13 ± 0.98, A: 1.59 ± 0.64, O: 2.01 ± 0.78) were significantly lower in A (*p* < 0.001, *p* < 0.001). The AUCs for distinguishing G, A, and O are shown in Table 2 and Figure 4. Overall, there was no significant difference between MET and rCBV. However, in G, rCBV showed a trend toward a higher AUC than MET (0.736 vs. 0.612, *p* = 0.08). Moreover, combining MET and rCBV improved performance over MET alone (*p* = 0.04) and was approximately comparable to rCBV alone (*p* = 0.21) in G. The absolute numbers of true positives, true negatives, false positives, and false negatives at the optimal cutoffs are summarized in Supplementary Table S1.

Among grade-based comparisons, both rCBV and the MET T/N ratio showed their highest values in grade 4. Only rCBV for grade 4 (AUC = 0.718) and the combined use of rCBV and MET (AUC = 0.796) exceeded 0.7. The combination significantly outperformed MET alone (AUC = 0.617, *p* = 0.016) but offered no additional benefit over rCBV alone (Supplementary Tables S2 and S3).

**Figure 5:**
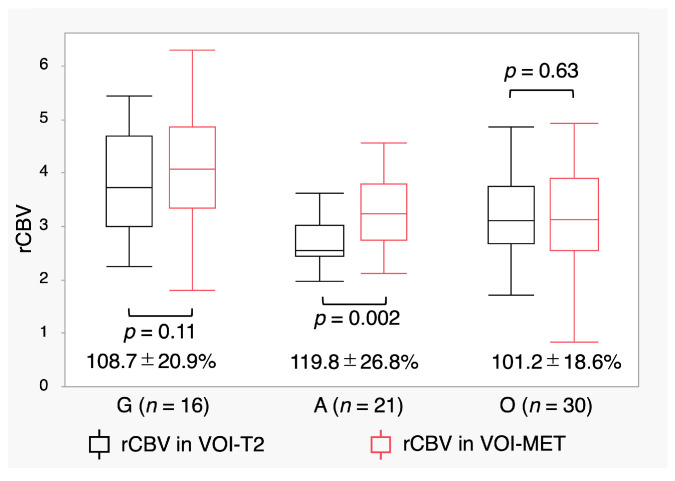
Relative cerebral blood volume (rCBV) in the volume of interest (VOI)-T2 and VOI- methionine (MET) in patients with MET accumulation. Box plots show rCBV values with MET accumulation. The increase ratio (%) of rCBV, expressed as rCBV in VOI-MET/rCBV in VOI-T2, is shown below the box plots. Lines within the boxes indicate the mean, whereas boxes represent the interquartile range.

Even when limited to 67 patients with MET accumulation, A had the lowest rCBV in VOI-T2 (G: 3.82 ± 1.0, A: 2.77 ± 0.68, O: 3.22 ± 0.93; *p* = 0.001). In contrast, O had the lowest rCBV in VOI-MET (G: 4.12 ± 1.18, A: 3.29 ± 1.00, O: 3.27 ± 1.10; *p* = 0.03). The rCBV in VOI-MET was significantly higher than that in VOI-T2 in A (*p* = 0.002) (Figure 5). The increased ratio of rCBV expressed as rCBV in VOI-MET/rCBV in VOI-T2 was the highest in A (*p* = 0.01) (Figure 5, Supplementary Table S4).

## 4. Discussion

The MET images in this study primarily reflect cell proliferation, tumor grade, and blood volume by excluding passive diffusion resulting from BBB disruption. Conversely, DSC-PWI calculates CBV based on time–concentration curves as the intravenously injected contrast agent passes through the vessels and brain tissue. It is known that leakage of contrast agents due to BBB disruption can reduce the accuracy of CBV measurements. However, in this study, we focused on non-contrast-enhanced lesions and did not need to address this issue. This approach allowed a more straightforward comparison of the characteristics of each glioma subtype.

First, in this study, ROC analyses of MET and rCBV for classifying G, O, and A showed no significant overall difference. However, for clinically important distinctions—particularly between G and grade 4—rCBV performed better than MET. Combining MET with rCBV improved discrimination compared to MET alone but offered no clear advantage over rCBV alone. This finding supports the utility of rCBV as a practical tool in clinical practice. Furthermore, MET uptake showed a moderate correlation with rCBV in G. These findings suggest that increases in rCBV may involve vascular mechanisms independent of MET. Unique vascular gene expression patterns (such as upregulation of angiopoietin-2 and the serpin family H) may be associated with the vascular remodeling characteristics of IDH-wild-type gliomas with elevated rCBV [25]. MET reflects increased cellular proliferation and metabolic activity, as previously reported [26]. This vascular phenotype may explain why rCBV outperformed MET in clinically relevant distinctions in G. In particular, some non-contrast-enhanced Gs showed little MET uptake, whereas rCBV may detect vascular remodeling that potentially precedes overt metabolic elevation. Because the WHO 2021 Classification defines all IDH-wildtype gliomas as grade 4, an increasing number of non-contrast-enhanced gliomas are now diagnosed as grade 4. These tumors are associated with poor prognosis and therefore require early imaging diagnosis. In this regard, rCBV appears effective in detecting early grade 4 gliomas, when blood volume begins to increase. MET accumulation is thought to rise as malignancy progresses. This process is associated with resulting in increased vascularity accompanied by endothelial proliferation and enhanced proliferative potential. Therefore, the effectiveness of rCBV and MET may depend on the stage of tumor growth.

In contrast, although both O and A harbor IDH mutations, O showed significantly higher rCBV than A. This may reflect prominent angiogenic branching and vascular hypertrophy in O, driven by potent pro-angiogenic mitogens, such as vascular endothelial growth factor and hypoxia-inducible factor-1α [27]. Therefore, O exhibits the unique feature of increased angiogenesis, leading to extensive vascular density and network formation [13,27]. The rCBV in VOI-T2 remained generally constant, regardless of MET accumulation, and the increase in rCBV within VOI-MET was less pronounced. The increase in rCBV observed in O was likely attributable to vascular proliferation rather than malignant cell proliferation. In A, by contrast, the absence of these vascular patterns resulted in a lower rCBV. Furthermore, MET-positive regions in A showed 119.8% higher rCBV than the surrounding tissue. These findings suggest that, in A, the observed correlation between rCBV and MET uptake may reflect a pathological feature in which angiogenesis progresses in parallel with increasing tumor malignancy.

Second, rCBV correlated with MET and predicted MET accumulation with a cutoff of 2.99 (AUC = 0.72). This method can be applied even in facilities without PET imaging equipment. MET uptake has previously been shown to have prognostic value [8]. It is conceivable that rCBV may serve as a surrogate biomarker for prognosis. However, this should be regarded as hypothesis-generating, and validation in larger cohorts is required.

Third, however, the intratumoral localization of MET accumulation varied by subtype. In A, MET uptake tended to colocalize with areas of elevated rCBV, whereas in O, rCBV was more uniformly distributed across the tumor, making it difficult to identify specific MET-positive regions. Previous studies have suggested the utility of rCBV as a surrogate for MET uptake across gliomas, but most combined contrast-enhanced and non-contrast-enhanced tumors, and different subtypes. When restricted to non-contrast-enhanced gliomas, our findings indicate that this surrogate relationship is less consistent. Thus, rCBV can provide guidance for identifying MET-positive regions, but its reliability remains dependent on tumor subtype and is limited. The number of MET-positive cases, particularly in G (16 cases), was relatively small, and this limitation should be considered when interpreting our findings.

The findings of this study have several important translational implications for clinical practice. Even in institutions without PET, identifying cases with likely MET accumulation or recognizing G at the non-contrast-enhanced stage may assist in determining the timing of surgery. Preoperative rCBV assessment can further support subtype-specific strategies: in A, rCBV-positive regions may guide biopsy targeting and more localized resection planning, whereas in G, rCBV evaluation may inform more extensive resection strategies and provide guidance for surgical planning even in cases without MET accumulation. The widespread accessibility of DSC-PWI compared with PET imaging makes these translational applications particularly relevant for routine clinical implementation. A similar situation has been reported in Parkinson’s disease, where PET can improve early diagnosis and accuracy when combined with MRI, but its limited availability restricts clinical use [28]. Although further studies are required to establish its full clinical utility, the widespread accessibility of DSC-PWI compared with PET imaging makes these translational applications particularly relevant for routine clinical implementation.

The significance of this study was to investigate the association between MET uptake and rCBV in a cohort of non-contrast-enhanced gliomas diagnosed according to the 2021 WHO Classification. This analysis suggests that, particularly in G, rCBV may capture unique vascular characteristics of IDH-wildtype tumors more directly than MET. Given the difficulty in diagnosing non-contrast-enhanced gliomas using a single modality, this study demonstrated that rCBV could be helpful as an ancillary diagnostic tool.

Limitations of this study include potential variability in ROI settings (e.g., whole vs. partial tumor, 2D vs. 3D analysis, mean vs. maximum values) and inter- and intra-observer differences, both of which may affect reproducibility. Furthermore, this was a retrospective single-institution study, and technical variability in rCBV across scanners, protocols, and software further limits generalizability. Finally, the results were based on [^11^C]-MET, which differs pharmacokinetically from more versatile [^18^F]-labeled tracers (e.g., FET, FDOPA), making direct transferability uncertain. Future multicenter prospective studies with standardized protocols are warranted.

## 5. Conclusions

rCBV and MET PET provide complementary mechanisms for evaluating non-contrast-enhanced gliomas. rCBV demonstrates diagnostic performance comparable to MET and can predict MET accumulation, suggesting that it may serve as a widely accessible ancillary tool in clinical practice. While its reliability varies by subtype, recognizing these differences may further enhance its clinical utility.

## Figures and Tables

**Table 1:** Baseline characteristics of non-contrast-enhanced adult-type diffuse gliomas divided by glioma subtypes.

	Total (*n* = 106)	G (*n* = 21)	A (*n* = 50)	O (*n* = 35)	*p*-Value
Age, mean	41.9 ± 12.4	49.5 ± 14.6	39.1 ± 12.5	41.5 ± 8.9	0.017
Male, *n*	57	9	30	18	0.393
Grade					<0.001
2, *n*	34	0	8	26	
3, *n*	50	0	41	9	
4, *n*	22	21	1	0	
MET accumulation, n	67	16	21	30	<0.001
MET T/N ratio	1.83 ± 0.79	2.13 ± 0.98	1.59 ± 0.64	2.01 ± 0.78	<0.001
rCBV in VOI-T2	2.98 ± 0.88	3.60 ± 1.0	2.60 ± 0.6	3.14 ± 0.89	<0.001

Data are shown as mean ± standard deviation, *p*-values for categorical variables were calculated using Pearson’s chi-squared test, G: glioblastoma isocitrate dehydrogenase (IDH)-wildtype, A: astrocytoma, IDH-mutant, O: oligodendrogliomas, IDH-mutant, and 1p/19q-codeleted, MET: methionine, T/N ratio: tumor-to-normal ratio, rCBV: relative cerebral blood volume, IDH: isocitrate dehydrogenase, VOI: volume of interest.

**Table 2:** ROC analysis of MET T/N ratio, rCBV, and combined MET T/N ratio + rCBV for predicting G, A, and O outcomes.

	Predictor	AUC (95% CI)	Cutoff	Sensitivity	Specificity
G	MET T/N ratio	0.612 (0.467–0.757)	>1.61	0.67	0.58
	rCBV	0.736 (0.607–0.865)	>3.29	0.67	0.81
	MET T/N ratio + rCBV	0.737 (0.606–0.868)	–	0.62	0.84
A	MET T/N ratio	0.712 (0.611–0.813)	<1.48	0.66	0.79
	rCBV	0.749 (0.655–0.842)	<3.41	0.94	0.45
	MET T/N ratio + rCBV	0.794 (0.707–0.880)	–	0.66	0.82
O	MET T/N ratio	0.659 (0.555–0.762)	>1.39	0.86	0.52
	rCBV	0.610 (0.496–0.724)	>2.56	0.8	0.45
	MET T/N ratio + rCBV	0.729 (0.630–0.827)	–	0.77	0.62

ROC: Receiver Operating Characteristic, MET T/N ratio: methionine tumor-to-normal ratio, rCBV: relative cerebral blood volume, G: glioblastoma isocitrate dehydrogenase (IDH)-wildtype, A: astrocytoma, IDH-mutant, O: oligodendrogliomas, IDH-mutant, and 1p/19q-codeleted, AUC: Area Under the Curve (95% Confidence Interval), Cutoff: Optimal cutoff value based on Youden index (MET T/N ratio + rCBV) is a predicted probability; threshold not applicable), Sensitivity: True positive rate at optimal cutoff, Specificity: True negative rate at optimal cutoff.

## Data Availability

The data that support the findings of this study are available from the corresponding author upon reasonable request.

## Supplementary Materials

The following supporting information can be downloaded at: https://www.mdpi.com/article/10.3390/jcm14196777/s1, Table S1: Confusion matrices (TP, TN, FP, FN) of MET T/N ratio, rCBV, and combined MET T/N ratio + rCBV for predicting G, A, and O outcomes. Table S2. Comparison of rCBV and MET T/N ratio across tumor grades. Table S3. ROC analysis of MET T/N ratio, rCBV, and combined MET T/N ratio + rCBV for predicting tumor grades 2, 3, and 4. Table S4. MET T/N ratio and rCBV in gliomas with MET accumulation.

## Abbreviations

The following abbreviations are used in this manuscript:

WHO	World Health Organization
MET	[^11^C]-methionine
PET	positron emission tomography
BBB	blood–brain barrier
rCBV	relative cerebral blood volume
DSC-PWI	dynamic susceptibility contrast-perfusion weighted imaging
MRI	magnetic resonance imaging
T2WI	T2-weighted imaging
T1WI	T1-weighted imaging
IDH	isocitrate dehydrogenase
VOI	volume of interest
T/N ratio	tumor-to-normal ratio
ROC	receiver operating characteristic
AUC	area under the curve
G	glioblastoma, IDH-wildtype
A	astrocytoma, IDH-mutant
O	oligodendroglioma, IDH-mutant and 1p/19q-codeleted
