# Supplementary material for: Evaluation of [11C]-Methionine Positron Emission Tomography and Cerebral Blood Volume Imaging in the Diagnosis of Non-Contrast-Enhanced Gliomas
Source: J Clin Med. 2025 Sep 25;14(19):6777. doi: 10.3390/jcm14196777 (PMC12525436; doi:10.3390/jcm14196777)
Supplement: Supplementary file 1 [file jcm-14-06777-s001.zip › jcm-3846559-supplementary.pdf]

Table S1. Confusion matrices (TP, TN, FP, FN) of MET T/N ratio, rCBV, and combined MET T/N ratio + rCBV for predicting G, A, and O outcomes.

	Predictor	TP	TN	FP	FN
G (*n* = 21)	MET T/N ratio	14	49	36	7
	rCBV	14	69	16	7
	MET T/N ratio + rCBV	13	71	14	8
A (*n* = 50)	MET T/N ratio	33	44	12	17
	rCBV	47	25	31	3
	MET T/N ratio + rCBV	33	46	10	17
O (*n* = 35)	MET T/N ratio	30	37	34	5
	rCBV	28	32	39	7
	MET T/N ratio + rCBV	27	44	27	8

TP: true positive; number of cases correctly classified as positive, TN: true negative; number of cases correctly classified as negative, FP: false positive; number of controls incorrectly classified as positive, FN: false negative; number of cases incorrectly classified as negative, G: glioblastoma isocitrate dehydrogenase (IDH)-wildtype, A: astrocytoma, IDH-mutant, O: oligodendrogliomas, IDH-mutant, and 1p/19q-codeleted, MET T/N ratio: methionine tumor-to-normal ratio, rCBV: relative cerebral blood volume.

Table S2. Comparison of rCBV and MET T/N ratio across tumor grades.

	Grade 2 (*n* = 34)	Grade 3 (*n* = 50)	Grade 4 (*n* = 22)	*p* value
MET T/N ratio	1.72 ± 0.74	1.79 ± 0.73	2.12 ± 0.96	0.238
rCBV	2.92 ± 0.63	2.76 ± 0.87	3.55 ± 1.01	0.002

Data are shown as mean ± standard deviation, MET T/N ratio: methionine tumor-to-normal ratio, rCBV: relative cerebral blood volume.

Table S3. ROC analysis of MET T/N ratio, rCBV, and combined MET T/N ratio + rCBV for predicting tumor grades 2, 3, and 4.

	Predictor	AUC (95% CI)	Cutoff	Sensitivity	Specificity	TP	TN	FP	FN
Grade2 (*n* = 34)	MET T/N ratio	0.545 (0.43-0.66)	< 1.82	0.74	0.44	25	32	40	9
	rCBV	0.532 (0.418-0.646)	> 2.58	0.76	0.47	26	34	38	8
	MET T/N ratio + rCBV	0.688 (0.581-0.795)	-	0.62	0.72	21	52	20	13
Grade 3 (*n* = 50)	MET T/N ratio	0.538 (0.426-0.65)	< 1.3	0.42	0.73	21	41	15	29
	rCBV	0.671 (0.568-0.775)	< 2.58	0.56	0.75	28	42	14	22
	MET T/N ratio + rCBV	0.696 (0.597-0.795)	-	0.88	0.43	44	24	32	6
Grade4 (*n* = 22)	MET T/N ratio	0.617 (0.477-0.757)	> 1.61	0.68	0.58	15	49	35	7
	rCBV	0.718 (0.589-0.847)	> 3.29	0.64	0.81	14	68	16	8
	MET T/N ratio + rCBV	0.796 (0.693-0.899)	-	0.86	0.67	19	56	28	3

ROC: Receiver Operating Characteristic, MET T/N ratio: methionine tumor-to-normal ratio, rCBV: relative cerebral blood volume. AUC: Area Under the Curve (95% Confidence Interval), Cutoff: Optimal cutoff value based on Youden index (MET T/N ratio + rCBV) is a predicted probability; threshold not applicable), Sensitivity: True positive rate at optimal cutoff, Specificity: True negative rate at optimal cutoff. TP: true positive; number of cases correctly classified as positive, TN: true negative; number of cases correctly classified as negative, FP: false positive; number of controls incorrectly classified as positive, FN: false negative; number of cases incorrectly classified as negative.

Table S4. MET T/N ratio and rCBV in gliomas with MET accumulation

	Total (n = 67)	G (n = 16)	A (n = 21)	O (n = 30)	*p*-value
Age, mean	42.7 ± 12.0	47.3 ± 14.9	38.8 ± 13.1	42.9 ± 8.7	0.148
Male, n	34	6	13	15	0.337
Grade					< 0.001
2, n	22	0	1	21	
3, n	28	0	19	9	
4, n	17	16	1	0	
MET T/N ratio	2.21 ± 0.77	2.13 ± 0.98	1.59 ± 0.64	2.01 ± 0.78	0.5
rCBV in VOI-T2	3.22 ± 0.95	3.82 ± 1.0	2.77 ± 0.68	3.22 ± 0.93	0.001
rCBV in VOI-MET	3.48 ± 1.13	4.12 ± 1.18	3.29 ± 1.00	3.27 ± 1.10	0.034
increase rate of rCBV	108.8 ± 23.1	108.7 ± 20.9	119.8 ± 26.8	101.2 ± 18.6	0.01

Data are shown as mean ± standard deviation, *p*-values for categorical variables were calculated using Pearson's chi-squared test, G: glioblastoma isocitrate dehydrogenase (IDH)-wildtype, A: astrocytoma, IDH-mutant, O: oligodendrogliomas, IDH-mutant, and 1p/19q-codeleted, MET: methionine, T/N ratio: tumor-to-normal ratio, rCBV: rela-tive cerebral blood volume, IDH: isocitrate dehydrogenase, VOI: volume of interest
